# Editorial: Zoonotic antimicrobial resistance and virulence: one health integrated approaches to monitor and reduce food chain hazards

**DOI:** 10.3389/fmicb.2024.1478509

**Published:** 2024-09-23

**Authors:** Ioana Cristina Marinas, Astrid Buica, Eliza Oprea, Mariana Carmen Chifiriuc

**Affiliations:** ^1^The Research Institute of the University of Bucharest, Bucharest, Romania; ^2^BiopharmSci R&D, AstraZeneca, Sweden; ^3^Department of Botany and Microbiology, Faculty of Biology, University of Bucharest, Bucharest, Romania; ^4^The Romanian Academy, Bucharest, Romania

**Keywords:** antimicrobial resistance, biosecurity, food safety, emerging food safety threats, One Health, toxicology, food safety risks

## Introduction

Antimicrobial resistance (AMR) and zoonotic pathogens' virulence pose public health, food safety, and global economic challenges, justifying the need for One Health (OH) approaches, acknowledging the interconnectedness of human, animal, and environmental sectors (Pitt and Gunn, 2024). Collaborative efforts across disciplines to bridge the gap between veterinary and human medicine, environmental science and food safety are needed to monitor and mitigate the risks of AMR and zoonotic infections in the food chain (Wielinga and Schlundt, 2014). Integrating OH principles into monitoring and reducing food chain hazards could lead to more effective interventions and policies to address these issues (European Food Safety Authority (EFSA) and European Centre for Disease Prevention and Control (ECDC), 2019; Compri et al., 2002).

Our Research Topic aimed to gather cutting-edge research that explores different aspects of zoonotic pathogens AMR and resistance using OH approaches. The articles accepted in this Research Topic cover several critical areas: (i) surveillance and monitoring through the use of advanced methods for detecting and tracking the virulence factors and AMR of zoonotic pathogens, as well as the role of whole genome sequencing and other molecular techniques in identifying resistance genes and monitor their spread (Andriyanov et al.; Gand et al.; Molina et al.; Morgan et al.); (ii) genetic and molecular mechanisms of AMR and virulence of zoonotic pathogens, and understanding of how these mechanisms evolve and spread within animal populations and the environment (Ma et al.); (iii) impact of zoonotic AMR on public health and food safety, revealed in epidemiological studies highlighting the relationship between animal breeding practices, the use of antibiotics and the emergence of resistant pathogens (Agusi et al.; Ouyang et al.).

Gand et al., in a metagenomic sequencing study, describe a promising method for pathogen detection and monitoring of AMR in food production environments based on the analysis of chicken fecal samples from a simulated-included community. The use of confidence levels for taxonomic identification and detection of AMR genes was proposed, as well as interpretation guidelines to facilitate data analysis with KMA (k-mer alignment tool), a popular bioinformatics software used for DNA sequence alignment and analysis. The ability of KMA to correlate detected AMR genes with the bacterial host chromosome within the same long sequence was also explored. The proposed methodology contributes to improving rapid diagnosis and surveillance of AMR pathogens and genes in food production environments, addressing the EU priorities (Gand et al.).

Molina et al. used long-read Oxford Nanopore MinION sequencing to determine the complete genome of a multi-drug-resistant *Escherichia coli* strain isolated from Ecuador from commercial cape gooseberry (*Physalis peruviana* L.) fruits, identify its serotype, multilocus sequence typing (MLST), virulence genes (*n* = 25), and AMR genes (*n* = 55) and demonstrated the necessity to carefully monitor these strains, especially in commercial settings (Molina et al.).

Another study (Agusi et al.) used standard microbiological methods and PCR to examine MDR *E. coli* prevalence, resistance, and virulence genes in poultry cloacal swabs from Jos (Nigeria). The prevalence of multi-drug-resistant *E. coli* was 45%, and these isolates harbored tetracycline, sulfonamide, ampicillin, and quinolone resistance genes as well as multiple virulence genes (e.g., *eae, stx, rfbe*, and *hlya*). These results suggest that poultry farming represents a significant source of environmental contamination with resistant genes and underlines the need for antimicrobial stewardship (Agusi et al.).

*Delftia tsuruhatensis*, an aerobic Gram-negative bacterium recently included in the category of human opportunistic pathogens, was isolated from raw bovine milk and studied by classical bacteriological methods as well as next-generation sequencing and comparative genomics (Andriyanov et al.). The strain proved resistant to 19 out of 23 tested antibiotics and harbored 27 virulence genes, mainly associated with motility, adherence, survival to stress, siderophore synthesis, and immunomodulation. Five prophage regions were identified in the genome of the mentioned strain, two of which are intact. The results of this study prove that raw milk can be a possible source and route of transmission/dissemination of this multi-resistant bacteria (Andriyanov et al.).

Morgan et al. conducted a comparative study of raw meat diets (RMDs) and non-raw meat diets (NRMD) products for dogs frequently bought in the UK, focusing on the top 10 brands, to determine the prevalence of *E. coli*, other *Enterobacteriaceae*, and *Salmonella* spp., as well as the prevalence of AMR and ESBL-producing *E. coli*. In the case of RMD samples, 39, 14, and 16% showed high counts of AMR, ESBL (more frequently carrying *blaCTX-M-15*), and 3GCR-*E. coli*. This study raises the need for strict hygiene measures for handling RMD products and increasing awareness of professionals and pet owners regarding the associated risks (Morgan et al.).

A study on nursery Hainan piglets investigated the effects of dietary supplementation with cecropin antimicrobial peptides (CAP, 500 mg/kg) on growth performance, diarrhea rate, and intestinal health (Ouyang et al.). The CAP-supplemented diet significantly improved average daily gain, reduced feed conversion ratio, and diarrhea rate. At the same time, this supplementation enhanced serum immunity and intestinal health, possibly due to increased villus height, better gut microbiota balance, and higher levels of immune proteins (Ouyang et al.).

Another study combines phenotyping and proteomics to examine how microbial population heterogeneity can lead to different stress responses and growth behaviors in *Listeria monocytogenes* (Ma et al.). Interestingly, a certain point mutation in the rpsU gene (*rpsU*^G50C^) has been shown to confer increased resistance to multiple stresses but a reduced maximum specific growth rate. The introduction of *rpsU*^G50C^ into the Δ*sigB* mutant for the sigma factor SigB, which regulates the general stress response, suggested the existence of an alternative signaling pathway for SigB activation in the *rpsU*^G50C^ mutants (Ma et al.).

Overall, this Research Topic of seven papers provides an excellent update in integrating efforts from various disciplines to monitor, understand, and mitigate the threats of AMR and the virulence of zoonotic agents, thereby protecting public health, ensuring food security, and promoting a more resilient global community.
